# Low-Dose ACTH Stimulation Test in Obesity: A Randomized Dose Assessment

**DOI:** 10.1155/2022/7860272

**Published:** 2022-11-19

**Authors:** Leonardo G. Mancillas-Adame, Adriana Sánchez-García, Rene Rodriguez-Gutierrez, Camilo Gonzalez-Velazquez, Fernando Javier Lavalle-Gonzalez, Jorge A. Zuñiga-Hernandez, Adriana Gabriela Rios-Ortega, José Gerardo González-González

**Affiliations:** ^1^Endocrinology Division, Department of Internal Medicine, University Hospital “Dr. Jose E. Gonzalez”, Universidad Autonoma de Nuevo Leon, Monterrey, Nuevo Leon, Mexico; ^2^Plataforma INVEST Medicina UANL-Ker Unit Mayo Clinic (Ker Unit México), Universidad Autónoma de NL, Monterrey 64460, Mexico; ^3^Knowledge and Evaluation Research Unit in Endocrinology, Mayo Clinic, Rochester, MN, USA

## Abstract

**Background:**

The short cosyntropin test is widely used for adrenal insufficiency screening and diagnosis. Lower cosyntropin doses may have greater sensitivity vs. the standard dose in detecting adrenal dysfunction. Obesity and overweight are increasing, impacting the clinical presentation of some diseases. Currently more than 50% of the subjects diagnosed with autoimmune adrenal insufficiency have a BMI greater than 25, and hence individuals living with overweight and obesity are more frequently requiring evaluation of the adrenal cortical function. Fixed-dose cosyntropin stimulation may not be appropriate for individuals with obesity.

**Objective:**

The primary objective was to compare cortisol response to a weight-adapted cosyntropin dose vs. a fixed low dose (1 *µ*g) and a more physiologically fixed dose (10 *µ*g).

**Methods:**

Twenty individuals with obesity and 20 age-matched healthy controls underwent in a randomized sequence at least one-week apart, to the The short cosyntropin test with three different doses, 0.2 *µ*g/kg of body weight, 1 or 10 *µ*g fixed dose stimuli. The assessment and data analysis were blinded to the individual and the investigator.

**Results:**

Cortisol response was reduced in the group with obesity with the 1 *µ*g fixed dose stimuli at 30 minutes (median, IQR) 649.6 *µ*g, 567.3–738.4 *µ*g for the control group vs. 568.6 *µ*g, 528.4–623.13 *µ*g, *p*=0.04; there was a lower cortisol peak at 60′ in all the three evaluated doses, with a dose-dependent trend. A weight-adapted cosyntropin dose of 0.2 *µ*g in obesity produces a similar response to the one observed in individuals without obesity. The 1 *µ*g ACTH test falls short on stimulating the cortisol adrenal response in individuals with obesity.

## 1. Introduction

Although there may be still some controversies on the dosing, the short cosyntropin test is widely used for adrenal insufficiency screening and diagnosis [1]. Classically, a single, 250 *µ*g, fixed dose was considered the standard stimulus [2]; however, reduced cosyntropin doses have been compared to the insulin tolerance test (ITT) providing similar peak serum cortisol responses [3, 4]. It is generally accepted that lower cosyntropin doses may have greater sensitivity vs. the 250 *µ*g test [5, 6]; a recent systematic review found similar diagnostic accuracy for low and standard doses [1]. However, the studies included in the systematic review did not take into account any of the baseline characteristics of the participants to individualize the ACTH dosing.

In the past, we proposed a more physiological dose to be used instead of 250 *µ*g. We compared 10 *μ*g vs. 250 *μ*g cosyntropin to evaluate the adrenocortical response in healthy individuals and in a variety of hypothalamic-pituitary-adrenal axis disorders. There were no differences in baseline or peak serum cortisol response in healthy subjects, but in some AI subjects, the results showed normal cortisol response during the standard test but abnormal cortisol response during the low-dose test. We also found better sensitivity with the low-dose vs. standard-dose cosyntropin stimulation test [7]. Graybeal and Fang studied the cortisol and ACTH responses to the insulin tolerant test, and they found that cortisol response to a 0.2 *µ*g/kg dose mimics the response to ITT [8]. Our reasoning at the moment was to test the typical individual being investigated for adrenal insufficiency, so, if planning to test a representative 50–70 kg of body weight individual, it was reasonable to use 10–14 *µ*g cosyntropin stimuli.

Obesity and overweight prevalence are rising around the globe, and the World Health Organization estimated a combined prevalence of overweight and obesity in adults by 2016 of 52% (39% overweight, 13% with obesity) [9]. Additionally, over the past four decades, the number of children and adolescents with obesity has risen more than tenfold [10]. Predictions have been made that if this trend continues, approximately 60% of the world's adult population could be either overweight or with obesity by 2030 [11]. This epidemiological revolution may impact the archetypal clinical presentation of some diseases, and one of those well described examples is type 1 diabetes, in which more than 30% of the subjects affected now are either overweight or living with obesity [12]. Adrenal insufficiency is not an exception and in a recent nationwide Swedish registry, Dalin et al. found more than 50% of the subjects diagnosed with autoimmune adrenal insufficiency having a BMI greater than 25 (36.9% between 25 and 30, 13.8% ≥ 30) [13]. Nowadays patients may present with overweight or obesity, not just because the increase trend describe above, but also because it can represent an earlier stage of the disease. So an underweight patient its not imprescindible to suspected adrenal insufficiency, since the typically wasted, underweight patient reported in the past, may represent a late stage of the disease [14].

Based on Graybeal's findings, suggesting that a weight-adjusted dose of 0.2 micrograms per kilo of body weight would elicit indistinguishable cortisol and cosyntropin response compared to the stress-induced ITT, we designed a blinded, randomized-sequence study aiming to compare the cortisol responses to 1 *µ*g and 10 *µ*g cosyntropin fixed doses and a weight-adjusted stimulation with 0.2 micrograms per kilo of cosyntropin in normal weight and individuals with obesity.

## 2. Participants and Methods

### 2.1. Participants

The study protocol was approved by the Institutional Research and Ethics Committee of “Dr. José Eleuterio González” University Hospital. Each volunteer gave their written informed consent before any study procedure. Twenty individuals with obesity (defined as a BMI equal or greater than 30) without comorbidities, between 18 and 60 years old, and 21 age-matched healthy controls with a BMI between 18.5 and 25 were included in the study. We excluded individuals with any endocrine disorder, use of glucocorticoids in the prior 24 months, and current use of drugs that may interfere with cortisol metabolism, transport, or the hypothalamus-pituitary-adrenal axis function. One subject on the healthy control group withdrew the consent before participating in any study procedure due to scheduling conflicts and was substituted with another age-matched volunteer. Individuals within the obesity group were invited to participate from the internal medicine, primary care, and endocrinology clinics of “Dr. José E. González” University Hospital once any medical condition other than obesity was ruled out.

### 2.2. Methods

Prior to study start, a blinded randomization procedure was designed to receive any of the three study doses (1 *µ*g, 10 *µ*g, or 0.2 mcg per kilogram of body weight) of cosyntropin, ACTH 1–24 (Cortrosyn®, Amphastar Pharmaceuticals Inc, Rancho Cucamonga, CA, USA) in a random sequence. Only the study coordinator (KCR) was aware of the testing dose allocation and was responsible for administering the blinded dose and blinding the blood samples. All cosyntropin stimulation tests were done at least seven days away from each other to avoid any carry-over effect. The cosyntropin preparation and dilution procedures were previously described [15].

Patients were instructed to be present at the laboratory facilities after an overnight fasting. Weight and height were measured using a calibrated scale (SOEHNLE®, Backnang, Germany) and between 0800 and 0900; while sitting in a blood drawing chair, an IV catheter (21-gauge vein needle set, BD Vacutainer ® Safety-Lok™ Blood Collection Set, Franklin Lakes, NJ, USA) was inserted into a forearm vein and a baseline sample was taken. The set was heparinized and subsequently, 0.2 *µ*g/kg, 10 or 1 of cosyntropin were injected as a bolus and blood samples were collected at +5, +10, +30 and + 60 minutes, eliminating the first 2 ml extracted to avoid dilution with the heparinized solution. The collected samples had the serum separated and preserved at −20°C in aliquots to be processed in batches to reduce the inter-assay variability. Cortisol was measured using a commercial electrochemiluminescence kit (Modular Analytics 6000, Roche/Hitachi Diagnostics, Mannheim, Germany). The intra-assay and inter-assay CV were 1.1% and 1.98%, respectively. The intra-assay and inter-assay CV were 8.7% and 9.8%, respectively.

### 2.3. Statistical Analysis

The database was blinded for the analysis regarding the dose administered and their sequence and unblinded for the authors only after the statistical analysis was finalized. Variables were tested for normality, using the Kolmogorov–Smirnov test. Median and interquartile range (IQR) are reported as central tendency and dispersion measures for continuous variables. Nominal variables are reported as proportions. The Mann–Whitney *U* test for independent samples was performed to test distributions of cortisol at baseline to compare across BMI categories. The Kruskal–Wallis test was done to compare the responses at 30 and 60 minutes to the three different doses of cosyntropin using as grouping variable the presence of obesity and finally to compare the three doses within the normal BMI and groups with obesity. Friedman's two-way analysis of variance by ranks was done. For all the inferential tests, a *p* value ≤0.05 was considered significant.

## 3. Results

### 3.1. Studied Population

Study population consisted of 40 participants, 20 in the group with obesity (oBMI) (median age: 24 years old, IQR 22–43) and 20 in the healthy control group (nBMI) (median age: 27 years old, IQR 22.2–35), *p* = 0.752. Median BMI was 33.9 kg/m^2^, IQR 31.1–39.1 in the oBMI and 23.5 kg/m^2^, IQR 21.6–24.5, in the nBMI, *p* < 0.001.

### 3.2. Cortisol Responses

**Figure 1:**
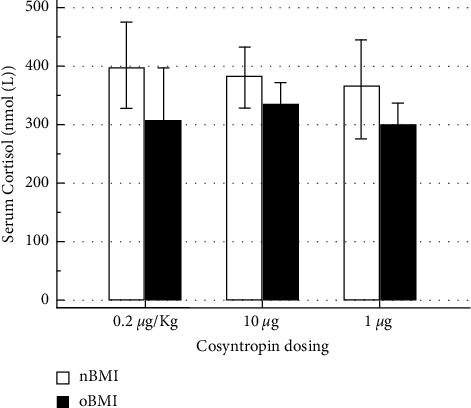
Basal serum cortisol (nmol/L) comparison across tests between normal weight (nBMI) and individuals with obesity groups (oBMI).

**Figure 2:**
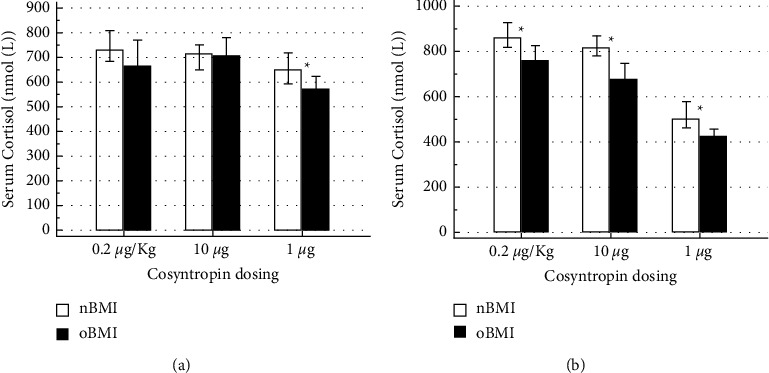
(a) Serum cortisol response to cosyntropin at +30 minutes (nmol/L). Results are presented as median, interquartile range. Kruskal–Wallis test for independent samples. ^*∗*^*p* value <0.05. (b) Serum cortisol response to ACTH at +60 minutes. Results are presented as median, interquartile range. Kruskal–Wallis test for independent samples. ^*∗*^*p* value <0.05.

Baseline serum cortisol was not different between nBMI and oBMI participants during either of the three stimulation tests (Figure 1).  Figure 2(a) presents the +30′ cortisol value in response to the three different stimulation doses used in the study. The oBMI group had a lower serum cortisol compared to the nBMI group, only during the 1 *µ*g stimulation test, with non-different responses to the weight adjusted or the 10 *µ*g cosyntropin fixed doses. Additionally when comparing the 30′ serum cortisol within the groups, it was lower in the 1 *µ*g test in both oBMI and nBMI groups. +60 minutes responses are shown in Figure 2(b). All study doses at +60′ exposed differences between oBMI and nBMI groups with lower responses in the former. The within group comparison, aligned with the 30′ results, showed a reduced response to the 1 *µ*g dose.

## 4. Discussion

In our study, otherwise healthy subjects with obesity had significantly lower serum cortisol than healthy subjects without obesity at 30 and 60 minutes after the application of a fixed dose of 1 *µ*g of cosyntropin as compared to the fixed 10 *µ*g cosyntropin dose. The lower response in subjects with obesity suggests that the stimulation caused by 1 *µ*g cosyntropin dose depends on subject's body weight. There was no difference at +30 minutes between the fixed 10 *µ*g cosyntropin dose compared to the weight-adjusted dose of 0.2 *µ*g/kg supporting the weight-related effect.

The relationship between cosyntropin dose, adrenal response, and body weight could be in part explained by the effect of fat distribution and obesity on HPA axis [16]. Roelfsema et al. reported that there may be a reduced adrenal sensitivity to cosyntropin in premenopausal women with obesity [17]. This altered adrenal response has been linked to body fat distribution. Pasquali et al. studied the adrenal response to two boli of 0.2 *µ*g/kg of BW of cosyntropin in healthy subjects with obesity reporting higher cortisol peak in subjects with abdominal obesity compared to peripheral obesity [18]. However, they did not find a correlation of stimulated cortisol and BMI or weight, and the study was done with a double stimulus. In our study, we did not find evidence of adrenal hyperresponsiveness to cosyntropin. We used BMI to define obesity finding a significant difference between groups in cortisol response at 30 and 60 minutes comparing low dose to the other studied doses without any significant differences in the baseline serum cortisol and randomizing the dose order to avoid any carry-over or preconditioning. Our results contrast with previously mentioned studies suggesting an effect of weight and BMI on adrenal response.

Our results have shown that cortisol response peak with 0.2 *µ*g/kg compared to 1 *µ*g was higher at 30 minutes in both studied groups. Cortisol values at 60 minutes were higher with 0.2 *µ*g/kg compared to the other doses. This finding suggests that weight-adjusted cosyntropin doses stimulate adrenal gland better than other doses. Our results contrast with Dickstein previous studies finding a maximal adrenal response with 1 *µ*g cosyntropin dose [19]. MacCario et al. studied lower doses, finding similar results with 1 *µ*g dose [20]. The anthropometric measurement adjusted to dose may influence adrenal response to the cosyntropin stimulation test. MacCario et al. studied body surface adjusted doses, showing no difference of cortisol response between lean women and women with obesity [20]; in their study, cortisol, aldosterone, and dehydroepiandrosterone responses to high, low, and very low cosyntropin doses in women with obesity overlapped with the age-matched lean control group responses. In contrast, Gonzalbez et al. did not find differences in the response to 1 *µ*g, 250 *µ*g, or ITT stimulation in the 30 min response vs. the peak response to the ITT [21]. We used a body weight dose approach, showing that this could be a more appropriate way to stimulate the cortisol response to cosyntropin in individuals living with obesity in whom adrenal insufficiency has to be ruled out. Further studies with more frequent and earlier sampling as well as comparison to ITT response are granted.

The strengths of our study include the design, a randomized clinical trial, the blinded analysis and the fact that we selected individuals without other comorbidities. Certainly, our study has some limitations, and we assessed the difference in response to different doses in healthy subjects with and without obesity. Our study findings would need to be reproduced and confirmed in individuals with suspected AI and overweight-obesity.

## 5. Conclusions

In conclusion, our findings indicate that 1 *µ*g cosyntropin dose to perform adrenal function test can lead to submaximal stimulation both in subjects with and without obesity and a weight-adjusted dose using 0.2 *µ*g/kg elicits a similar response in both the oBMI and nBMI groups. An insufficient adrenal stimulation in the context of a stimulation test might have diagnostic and therapeutic consequences which need to be confirmed in new studies. On the basis of our findings in AI, the performance of other dynamic endocrine testing should be evaluated in individuals living with obesity.

## Data Availability

The data that support the findings of this study are available from the corresponding author upon reasonable request.
